# Real-time fluorescent multiple cross displacement amplification for rapid and sensitive *Mycoplasma pneumoniae* detection

**DOI:** 10.3389/fcimb.2024.1423155

**Published:** 2024-08-08

**Authors:** Fei Xiao, Yu Zhang, Wenjian Xu, Jin Fu, Xiaolan Huang, Nan Jia, Chunrong Sun, Zheng Xu, Baoying Zheng, Juan Zhou, Yi Wang, Lihui Meng

**Affiliations:** ^1^ Experiment Research Center, Capital Institute of Pediatrics, Beijing, China; ^2^ Laboratory Center, Children’s Hospital Affiliated to the Capital Institute of Pediatrics, Beijing, China; ^3^ Respiratory Medicine, Children’s Hospital Affiliated to the Capital Institute of Pediatrics, Beijing, China; ^4^ Department of Infectious Diseases, Children’s Hospital Affiliated to Capital Institute of Pediatrics, Beijing, China

**Keywords:** *Mycoplasma pneumoniae*, real-time detection, multiple cross displacement amplification, restriction endonuclease, rapid diagnosis

## Abstract

*Mycoplasma pneumoniae* is a significant pathogen responsible for community-acquired pneumonia, predominantly affecting children and adolescents. Here, we devised a rapid method for *M. pneumoniae* that combined multiple cross displacement amplification (MCDA) with real-time fluorescence technology. A set of ten primers, which were specifically designed for *M. pneumoniae* detection, were employed in a real-time fluorescence MCDA reaction. Of these, one primer incorporated a restriction endonuclease recognition sequence, a fluorophore, and a quencher, facilitating real-time fluorescence detection. The real-time (RT)-MCDA reactions were monitored in a simple real-time fluorescence instrument and conducted under optimised conditions (64°C for 40 min). The detection limit of the *M. pneumoniae* RT-MCDA assay for genomic DNA extracted from *M. pneumoniae* culture was down to 43 fg/µl. This assay accurately identified *M. pneumoniae* strains without cross-reacting with other bacteria. To validate its practical application, we tested the *M. pneumoniae* RT-MCDA assay using genomic DNA extracted from clinical samples. The assay’s detection capability proved comparable with real-time PCR, MCDA-based biosensor detection, and visual inspection under blue light. The entire process, including rapid DNA extraction and real-time MCDA detection, was completed within 1 h. Overall, the *M. pneumoniae* RT-MCDA assay reported here is a simple and effective diagnostic tool for rapid *M. pneumoniae* detection, which holds significant potential for point-of-care testing and in resource-limited regions.

## Introduction

*Mycoplasma pneumoniae* is a prevalent cause of community-acquired pneumonia (Hammerschlag, 2001; Lee, 2008; Kumar and Kumar, 2023). This infectious agent can affect individuals of all ages but is most common among children and adolescents, with its incidence rising annually (Chang et al., 2014; Chen et al., 2024; Liu et al., 2024). Infections with *M. pneumoniae* typically manifest with respiratory symptoms but it can also lead to complications outside the respiratory system, such as cardiovascular and neurological complications (Kumar and Kumar, 2023). The clinical signs of *M. pneumoniae* infection are non-specific and coupled with diagnostic complexities, making early and rapid identification challenging (Hammerschlag, 2001; Kumar and Kumar, 2023). Therefore, improving the accuracy of *M. pneumoniae* detection is particularly important for clinical diagnosis and management.

There is no significant difference in clinical symptoms and imaging between *M. pneumonia* infection-associated pneumonia and pneumonia caused by other pathogens. Thus, the identification and diagnosis of *M. pneumoniae* infection mainly relies on laboratory diagnosis (Hammerschlag, 2001; Daxboeck et al., 2003; Kumar and Kumar, 2023). Currently, the main techniques for detecting *M. pneumoniae* are the culture-based method, serological tests, and polymerase chain reaction (PCR) (Kumar and Kumar, 2023). The culture-based method is regarded as the “gold standard” for confirming *M. pneumoniae* infections (Daxboeck et al., 2003; Chang et al., 2014; Kumar and Kumar, 2023). However, it is time-consuming (2–6 weeks for results), labour-intensive, and costly, limiting its practical use in clinical settings (Chang et al., 2014; Kumar and Kumar, 2023). Serological testing is commonly used due to its ease of use (Daxboeck et al., 2003; Chang et al., 2014). However, challenges in producing highly specific *M. pneumoniae* antibodies, due to numerous stop codons in the *M. pneumoniae* gene sequence and difficulties in recombinant antigen production via prokaryotic expression, affect the sensitivity and specificity of this method. Factors such as patient age, immune status, and the quality of test kits also impact results (Daxboeck et al., 2003; Chang et al., 2014). In addition, the requirement for paired serum samples taken 1–2 weeks apart makes serological testing less accessible and generally retrospective (Chang et al., 2014). A PCR-based detection assay is emerging as a rapid and reliable diagnostic method, often regarded as the new “gold standard” for detecting *M. pneumoniae* due to its high sensitivity and specificity (Chang et al., 2014; Park, 2022). However, its widespread adoption is hampered by the need for sophisticated equipment and skilled operators, particularly in primary healthcare settings (Chang et al., 2014; Park, 2022). To address these limitations presented by PCR-based methods, various isothermal amplification techniques have been developed since the early 1990s as alternatives to PCR (Zhao et al., 2015; Park, 2022; Srivastava and Prasad, 2023). These methods allow for the rapid and efficient amplification of nucleic acids at a constant temperature, offering advantages such as lower equipment costs, quicker results, simpler procedures, and greater specificity and sensitivity, making them suitable for immediate clinical use (Wang et al., 2015, 2016).

In recent years, the use of multiple cross displacement amplification (MCDA) technology within the realm of isothermal amplification has proven effective for detecting numerous pathogens (Zhao et al., 2019; Li et al., 2020; Jiang et al., 2022; Jia et al., 2023; Zhou et al., 2023). MCDA is a novel amplification strategy based on isothermal chain displacement polymerisation reactions, in which a set of 10 specific primers is designed for each target, spanning 10 different regions of the target sequence, achieving sensitivity in the femtogram range (Wang et al., 2015). At the operational temperatures (58°C–70°C), the dynamic interaction between primer-template hybridisation facilitates the annealing of a high concentration of primers onto the DNA template without requiring a denaturation phase, thus initiating synthesis (Wang et al., 2015). This leads to exponential amplification of the DNA products through chain displacement reactions. The MCDA reactions can be monitored through three primary methods: colorimetric analysis, agarose gel electrophoresis, and real-time turbidimetry. Therefore, the MCDA technique not only offers rapid results, high specificity, and sensitivity, but also has the added benefits of low equipment demands and simplicity in execution.

In this study, we successfully incorporated MCDA technology into the real-time PCR platform, creating a novel real-time MCDA detection method specifically tailored for *M. pneumoniae*. This approach enables direct monitoring of fluorescence signals and allows results to be read directly through the instrument without having to open the reaction tube, significantly reducing the risk of contamination. This rapid, sensitive, specific, and contamination-free method proves highly effective in diagnosing *M. pneumoniae* infection. During the establishment of this technique, we also assessed its clinical accuracy in detecting *M. pneumoniae* infection. The results of our study indicate that *M. pneumoniae* RT-MCDA is particularly useful in regions lacking dedicated product testing laboratories.

## Materials and methods

### Reagents and instruments

A DNA Isothermal Amplification Kit, biotin-14-dCTP, visual detection reagent (VDR), and a nanoparticle-based lateral flow biosensor (LFB) were obtained from Huidexin Biotech Co., Ltd (Tianjin, China). Restriction endonuclease (*Nb*.*BsrDI*) was purchased from New England BioLabs (Beijing, China). A nucleic acid extraction kit was obtained from Beijing Transgen Biotech Co., Ltd (Beijing, China) and real-time PCR kits were procured from HuNan SX Bio-Tech Co., Ltd (HuNan, China). The BluSight Pro (GD50502) was obtained from Manod Biotech. Co, Ltd (Suzhou, China). The real-time turbidimeter (LA-320C) was purchased from Eiken Chemical Co., Ltd, Japan. A fluorescence quantitative PCR instrument ABI 7500 was purchased from Applied Biosystem Inc., USA.

### Primer design

**Figure 1:**
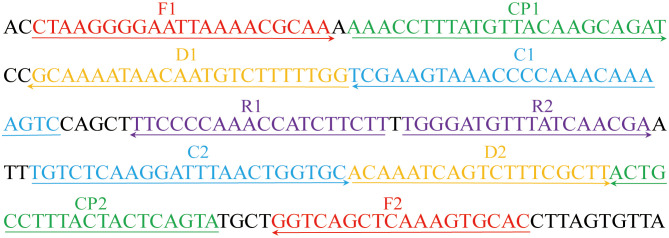
Primers for the *CARDS* toxin gene used in the *M. pneumoniae* RT-MCDA assay in this study. The location of the primer sequences used in this study on the targeting *CARDS* toxin gene of *M. pneumoniae*. Right and left arrows show sense and complementary sequences, respectively. The coloured text indicates the position of the primers, including two displacement primers (F1 and F2), two cross primers (CP1 and CP2), and six amplification primers (C1, C2, D1, D2, R1, and R2).

A set of ten primers targeting the community-acquired respiratory distress syndrome (CARDS) toxin gene (LR214945.1) of *M. pneumoniae* were designed using primer premier 5.0 (https://primerexplorer.jp). The primer set includes two displacement primers (F1 and F2), two cross primers (CP1 and CP2), and six amplification primers (C1, C2, D1, D2, R1, and R2). The amplification primer D1 used in the *M. pneumonia* MCDA-LFB assay (termed D1^#^) was modified by assigning a fluorophore (FAM) at the 5' end, whereas the amplification primer C1 employed in the *M. pneumoniae* RT-MCDA assay (termed C1*) was modified by additionally adding a short sequence (Ss, TGCAATG) at the 5′ end and assigning a fluorophore and black hole quencher 1 (BHQ1) at the 5′ end and the middle of new primer. Primers and labelled primers used in this study were synthesised by AoKe Biotech Co., Ltd (Beijing, China). The sequences, location, and the modification of the primers are shown in **Table 1** and **Figure 1**.

**Table 1:** The primers used in the study.

Gene	Primer^a^	Sequence and modifications (5′-3′)	Length^b^
CARDs toxin	F1	CTAAGGGGAATTAAAACGCAA	21nt
	F2	GTGCACTTTGAGCTGACC	18nt
	CP1	GACTTTTGTTTGGGGTTTACTTCGA AACCTTTATGTTACAAGCAGAT	47mer
	CP2	TGTCTCAAGGATTTAACTGGTGCATA CTGAGTAGTAAAGGCAGT	44mer
	C1	GACTTTTGTTTGGGGTTTACTTCGA	25nt
	C2	TGTCTCAAGGATTTAACTGGTGC	23nt
	D1	CCAAAAAGACATTGTTATTTTGC	23nt
	D2	ACAAATCAGTCTTTCGCTT	19nt
	R1	AAGAAGATGGTTTGGGGAA	19nt
	R2	TGGGATGTTTATCAACGA	18nt
	D1^#^	FAM-CCAAAAAGACATTGTTATT TTGC	23nt
	C1*	FAM-TGCAATG-GACT(BHQ1)TTTGTTTGGGGTTTACTTCGA	25nt

a F1 and F2, displacement primers; CP1 and CP2, cross primers; C1, C2, D1, D2, R1, and R2, amplification primers; C1*, 5′-labelled with FAM and BHQ1 when used in an M. pneumoniae RT- MCDA assay; D1^#^, 5′-labelled with FAM when used in an M. pneumoniae-MCDA-LFB assay.

b nt, nucleotide; mer, monomeric unit.

### DNA preparation

Genomic DNA of all the strains was extracted and purified using an EasyPure^®^ Genomic DNA kit (TransGen Biotech, Beijing, China) according to the manufacturer’s instructions. Purity and concentration were determined using a Nanodrop 2000 (Thermo Fisher, Waltham, MA, USA) at A260/280. Before usage, the extracted DNA was kept at −20°C.

### Standard *M. pneumoniae* -MCDA-LFB reaction

The volume of the MCDA reaction system was 25 μl, containing 12.5 μl of 2 × isothermal reaction buffer, 0.1 μmol/L each of displacement primers (F1 and F2), 0.4 μmol/L each of cross primers (CP1 and CP2), 0.2 μmol/L each of amplification primers [C1, C2, D1^#^(FAM labelled), D2, R1, and R2], 1.0 μl of *Bst* 2.0 DNA polymerase (8 U), 0.5 μl of biotin-14-dCTP, and 1 μl of DNA template from *M. pneumoniae* pure culture or 5 μl from clinical samples. In addition, 1 µl of DNA template of *Klebsiella pneumoniae* was employed as a negative control, and 1 µl of double distilled water (DW) was used as a blank control. The reaction mixtures were incubated at 64°C for 1 h using a real-time turbidimeter or traditional PCR instrument. The results were monitored using a real-time turbidimeter, VDR, and LFB test. For real-time turbidimeter, reaction with turbidity >0.1 was considered as positive, otherwise as negative. For VDR, reaction with a light blue colour was deemed as positive, while the colourless one was negative. For result interpretation by LFB, 5 µl of MCDA reaction products were added to the sample pad of LFB, followed by 100 μl of running buffer [10 mM PBS (PH 7.4) with 1% Tween 20]. The results were indicated within 2 min, with two red lines at the control line (CL) and testing line (TL) representing a positive result and one red line at the CL representing a negative result. In parallel, results of the *M. pneumoniae* MCDA reaction were real-time monitored by real-time turbidimeter and visually inspected by naked eye with VDR.

### Standard *M. pneumoniae* RT-MCDA reaction

The RT-MCDA assay was conducted in a 25 μl reaction system consisting of 12.5 μl of buffer, 0.1 μmol/L each of displacement primers (F1 and F2), 0.4 μmol/L each of cross primers (CP1and CP2), 0.2 μmol/L each of amplification primers [C1^*^ (restriction endonuclease recognition sequence, FAM and BHQ1 labelled), C2, D1, D2, R1, and R2], 1.0 μl of Bst DNA polymerase (8 U), 1.0 μl of *Nb*.*BsrDI* restriction endonuclease, and 1 μl of template from pure culture (5 μl from the clinical sample). The reaction was carried out using real-time PCR instruments for 40 cycles with a setting mode of 5 s at 64°C for reaction and 55 s at 64°C for fluorescence monitoring. Reaction with apparent fluorescence generation indicated a positive result, while that with trace or none fluorescent signal implied a negative one. The positive threshold value was obtained by testing plenty of positive, negative, and blank reactions.

### Optimisation of the *M. pneumoniae* RT-MCDA assay

The optimal reaction temperature for the *M. pneumoniae* MCDA reaction was determined by performing an *M. pneumoniae* MCDA reaction at temperatures from 60 to 67°C with a 1°C interval for 40 min followed by incubation at 85°C to stop the reaction. The amplification process was real-time monitored using a real-time turbidimeter. DNA templates from *M. pneumoniae* were used as a positive control and DW as a blank control. The temperature generating the best reaction performance was considered as the optimal one. Meanwhile, assays with different reaction times from 10 min to 40 min (with 10 min intervals) were conducted to determine the optimal reaction time. The shortest time required for the MCDA reaction was regarded as the optimal reaction time. All the tests were repeated three times. The obtained optimum reaction temperature and time were employed for the following tests.

### Sensitivity and specificity evaluation of the *M. pneumoniae* RT-MCDA assay

To determine the sensitivity of the *M. pneumoniae* RT-MCDA assay, DNA templates from cultured *M. pneumoniae* were serially diluted to 4.3 ng/μl, 430 pg/μl, 43 pg/μl, 4.3 pg/μl, 430 fg/μl, 43 fg/μl, and 4.3 fg/μl. Accordingly, 1 μl or 2 μl each of the serial templates was added into the *M. pneumoniae* RT-MCDA reaction systems to test the limit of detection (LoD) of the assay. Each reaction was repeatedly tested in triplicate. For the specificity assessment of the *M. pneumoniae* RT-MCDA assay, DNA templates from three *M. pneumoniae* strains and 29 non-*M. pneumoniae* strains (**Table 2**) were examined by this assay with three replicates. Similarly, all the samples were tested by the MCDA-LFB assay for comparison.

**Table 2:** Strains for specificity confirmation of *M. pneumoniae* RT-MCDA assay.

Pathogen	Strain no. (source of the strains)^a^	No. of strains	*M. pneumoniae* real-time MCDA^b^
*Mycoplasma pneumonia*	Isolated strains (CDC)	3	P
*Acinetobacter baumannii*	Isolated strains (CIP)	1	N
*Bacillus cereus*	Isolated strains (CIP)	1	N
*Citrobacter* spp.	Isolated strains (CIP)	1	N
*Corynebacterium striatum*	Isolated strains (CIP)	1	N
*Enteroinvasive Escherichia coli*	Isolated strains (CIP)	1	N
*Enterotoxigenic Escherichia coli*	Isolated strains (CIP)	1	N
*Haemophilus influenza*	Isolated strains (CIP)	2	N
*Klebsiella pneumonia*	Isolated strains (CIP)	2	N
*Listeria innocua*	Isolated strains (CIP)	1	N
*Listeria monocytogenes*	Isolated strains (CIP)	1	N
*Monilia albican*	Isolated strains (CIP)	1	N
*Moraxella catarrhalis*	Isolated strains (CIP)	1	N
*Mycobacterium tuberculosis*	Isolated strains (CIP)	1	N
*Neisseria meningitides*	Isolated strains (CIP)	1	N
*Nocardia asteroids*	Isolated strains (CIP)	1	N
*Pseudomonas aeruginosa*	Isolated strains (CIP)	1	N
*Shigella sonnei*	Isolated strains (CIP)	1	N
*Staphylococcus amber*	Isolated strains (CIP)	1	N
*Staphylococcus haemolyticus*	Isolated strains (CIP)	1	N
*Steptococcus salivarius*	Isolated strains (CIP)	1	N
*Streptococcus aureus*	Isolated strains (CIP)	1	N
*Streptococcus pneumoniae*	Isolated strains (CIP)	2	N
*Streptococcus pyogenes*	Isolated strains (CIP)	1	N
*Mycoplasma genitalium*	Isolated strains (CIP)	1	N
*Mycoplasma genitalium*	Isolated strains (CIP)	1	N
*Mycoplasma urealyticum*	Isolated strains (CIP)	1	N

**^a^** CIP, Capital Institute of Pediatrics; CDC, Chinese Center for Disease Control and prevention.

**^b^** P, positive; N, negative.

### Clinical application assessment of the *M. pneumoniae* RT-MCDA assay

For evaluation of the clinical feasibility, DNA templates extracted from 48 sputum samples were retrospectively tested by the *M. pneumoniae* RT-MCDA assay and MCDA-LFB test. Particularly, 1 µl, 3 µl, 5 µl, and 7 µl of clinical sample template were examined separately to identify the appropriate volume for clinical evaluation. The 48 samples were collected from children hospitalised in the Children’s Hospital Affiliated to Capital Institute of Pediatrics from 12 October 2023 to 20 December 2023 and had been examined using the real-time PCR method for clinical diagnosis. All the samples were obtained with informed consent signed by the guardians of the participants.

## Results

### Principle of the *M. pneumoniae* RT-MCDA assay

**Figure 2:**
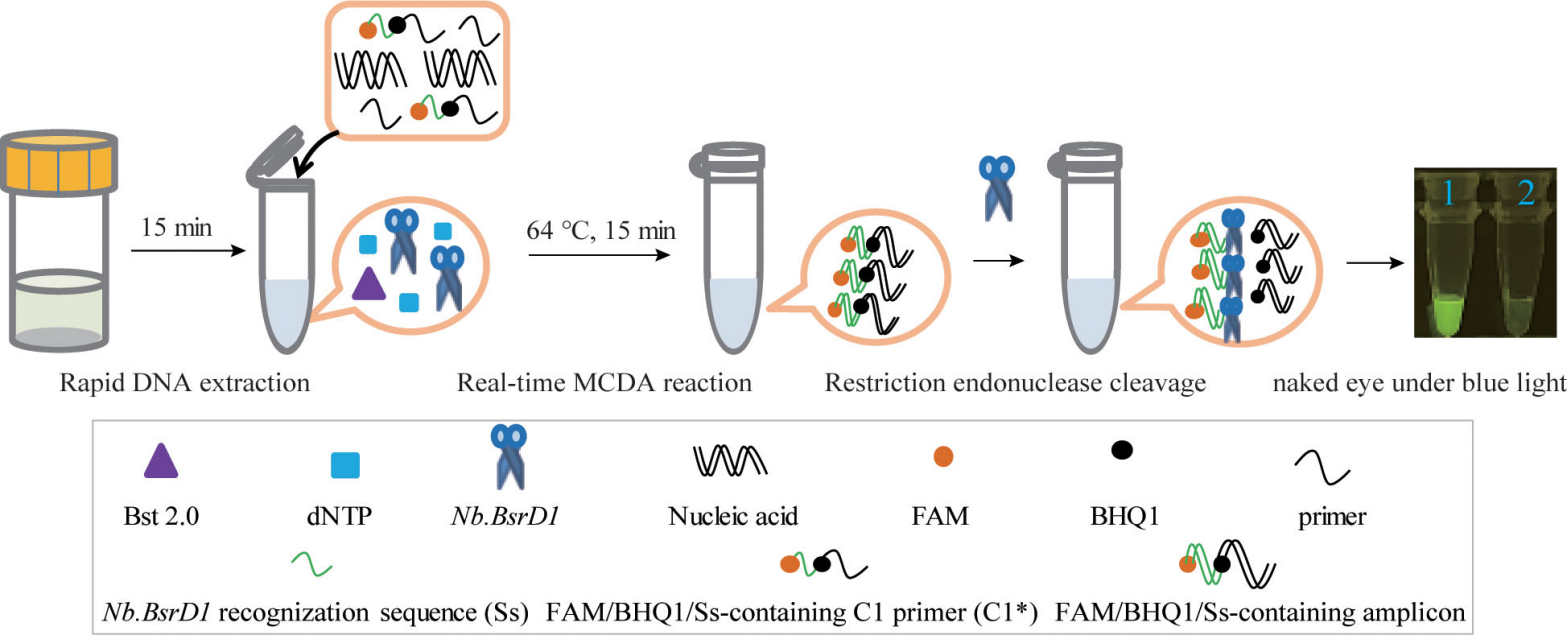
Schematic illustration of the *M. pneumoniae* RT-MCDA assay. In the *M. pneumoniae* RT- MCDA system, the primer C1* was modified by adding a short sequence (TGCAATG) at 5′ end that could be recognised by restriction endonuclease *Nb.BsrDI*. When reacted with C1*, the *M. pneumoniae* real-time MCDA system generated plenty of Ss-containing target amplicons that were then cleaved by restriction endonuclease *Nb.BsrDI*, which could separate the fluorophore FAM from BHQ1, resulting in the emission of fluorescence signals that could be visually inspected with the naked eye under blue light, in addition to a real-time PCR instrument.

The *M. pneumoniae* RT-MCDA assay was devised by combining an MCDA reaction with restriction endonuclease cleavage, which will generate fluorescent readouts for result interpretation. As illustrated in **Figure 2**, using primers (C1* in this study) adding a short sequence 5′-TGCAATG-3′, which could be specifically recognised and cleaved by restriction endonuclease *Nb.BsrDI*, the RT-MCDA reaction will produce plenty of products containing the *Nb.BsrDI* recognition sequence. Once the generated products were cleaved by *Nb.BsrDI*, fluorescent signals were yielded due to the separation of FAM and BHQ1, which flanked the *Nb.BsrDI* recognition sequence. It is worth mentioning that owing to the addition of the short sequence (Ss, TGCAATG) at the 5′ end of the amplification primer C1 (C1*), *Nb.BsrDI* accurately identified the target double-stranded DNA sequences and cut them to generate fluorescence signals, which will not be interfered with by the excess (single-stranded) primer cleavage. The fluorescent signals could be visually inspected with the naked eye under blue light in addition to a real-time PCR instrument. Thus, the presence or absence of target pathogen will be reported only with the naked eye rather than complicated instruments.

**Figure 3:**
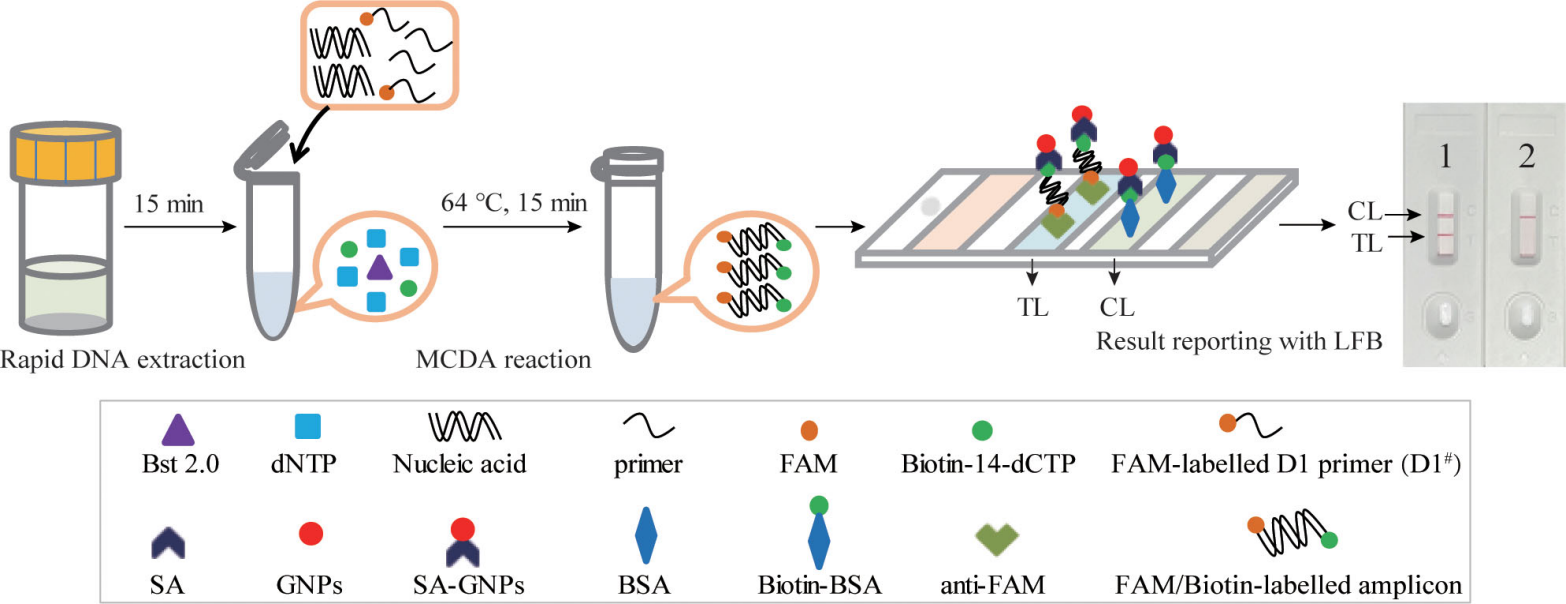
Schematic illustration of the *M. pneumoniae*-MCDA-LFB assay. In the *M. pneumoniae* -MCDA-LFB system, anti-FAM immobilised on LFB can capture the double-labelled amplicon (produced by FAM-labelled amplification primer D1^#^ and biotin-14-dCTP) in the reaction and is visualised by the reaction of biotin and SA-GNPs (dark red), resulting in a red line occurring in the TL region of the LFB. The remaining SA‐GNPs were captured by the immobilised biotin‐BSA at the CL region, leading to a red line occurring in the CL region, which indicated the effectiveness of the LFB.

In this study, an *M. pneumoniae*-MCDA-LFB assay was conducted in parallel for comparison. The principle of the MCDA-LFB assay is shown in **Figure 3**. Unlike the RT-MCDA assay, the MCDA-LFB assay was devised on the basis of the MCDA reaction and LFB detection. Using a FAM-labelled primer and biotin-14-dCTP, the MCDA reaction could produce abundant FAM/biotin-labelled amplicons, which could be transformed into a colorimetric readout with LFB. Particularly, the immobilised anti-FAM of LFB captured FAM-containing target amplification products and SA-GNPs (streptavidi-coated dyed polymer nanoparticles) enabled products containing biotin to be visualised, resulting in a red line occurring in the TL region of LFB. The remaining SA-GNPs were captured by the biotinylated bovine serum albumin (biotin-BSA) in the CL region, resulting in a red line in the CL region, indicating the effectiveness of the LFB (**Figure 3**).

### Effectiveness of the primer set for the *M. pneumoniae* RT-MCDA assay

**Figure 4:**
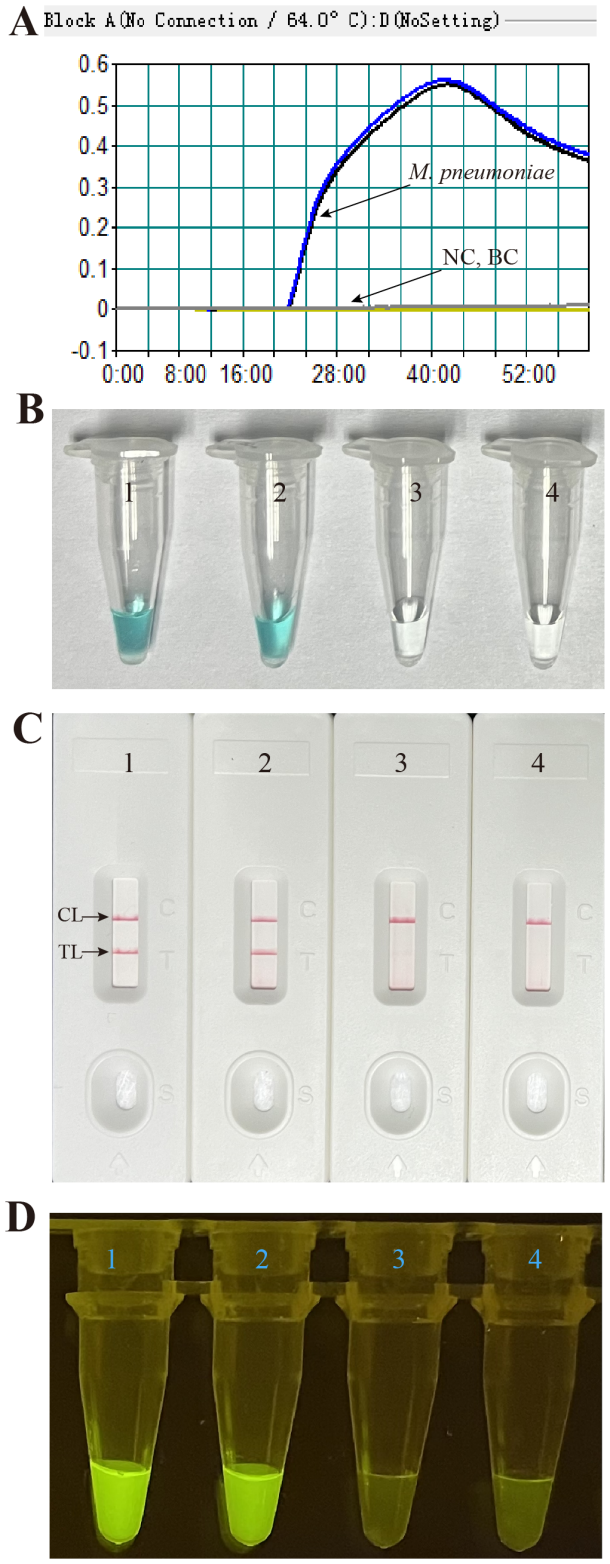
Effectiveness of the primer set for the *M. pneumoniae* RT-MCDA reaction. The DNA templates extracted from *M. pneumoniae* strains were effectively amplified with MCDA reactions at 64°C for 1 h, and there was no obvious amplification in the negative and blank controls. The products were indicated by real-time turbidity **(A)**, the VDR **(B)**, and the nanoparticle-based LFB test **(C)**, and observed with the naked eye under blue light **(D)**. Tubes/strips 1–2 represent DNA templates extracted from *M. pneumoniae* strains. Tube/strip 3 represents DNA template extracted from a *Klebsiella pneumoniae* strain, Tube/strip 4 represents the blank control. CL, control line; TL, test line.

The effectiveness of the primer set (**Figure 1**, **Table 1**) for the *M. pneumoniae* MCDA assay was confirmed by performing an MCDA reaction with the selected primer set and monitoring results using three methods (real-time turbidity, VDR, and LFB). Using a real-time turbidimeter, the positive reaction (the reactions of *M. pneumoniae*) displayed a sharp increase in turbidity, whereas the negative (the reaction of *K. pneumoniae*) and blank control (the reaction of DW) reactions did not generate any turbidity (**Figure 4A**). Using VDR, the colour of the positive control changed into light green, whereas the others were colourless (**Figure 4B**). With LFB, two visible red lines in the CL and TL regions were observed with the positive control products, whereas only one line in CL was observed when the negative and blank control products were examined (**Figure 4C**). Thus, the primer set was determined to establish the *M. pneumoniae* RT-MCDA assay.

Subsequently, the primer set was used for *M. pneumoniae* RT-MCDA assay confirmation. As shown in **Figure 4D**, positive reactions released fluorescent signals that showed an obvious fluorescence value in the real-time PCR platform and a light yellow colour under blue light, whereas the negative and blank controls did not present a fluorescent signal either in the real-time PCR platform or under blue light. These data indicated that the selected primer set was suitable for the *M. pneumoniae* RT-MCDA assay and *M. pneumoniae* detection.

### Optimal reaction condition for the *M. pneumoniae* RT-MCDA assay

**Figure 5:**
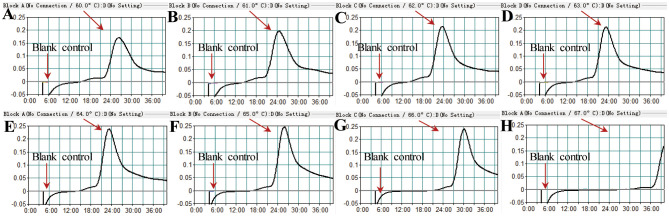
Temperature optimisation for the *M. pneumoniae* RT-MCDA assay. MCDA reactions detecting *M. pneumoniae* were conducted using a real-time turbidimeter. Turbidity >0.1 was considered a positive result. The kinetic curves at different temperatures ranging from 60 to 67°C **(A–H)** were acquired, showing that 64°C was the optimal temperature.

**Figure 6:**
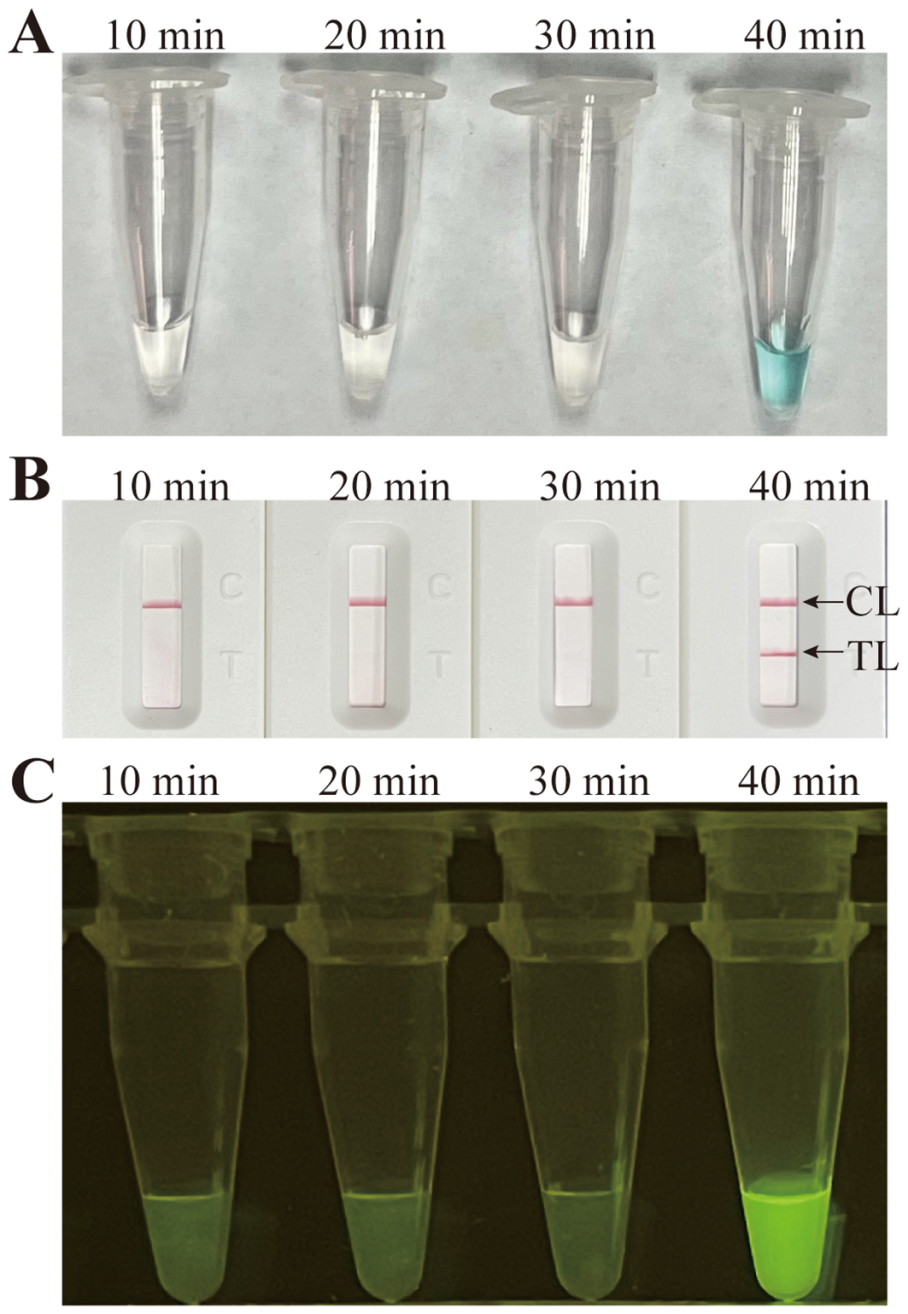
Time optimisation for the *M. pneumoniae* RT-MCDA assay. The standard MCDA reaction with four distinct times, 10 min, 20 min, 30 min, and 40 min, was tested and analysed by VDR **(A)**, LFB **(B)**, and the naked eye under blue light **(C)** at the optimal temperature of 64°C. The LoD level of *M. pneumoniae* genomic DNA could be detected only after amplification for 40 min. Strips/tubes 1–7, DNA templates extracted from serial dilutions (4.3 ng–4.3 fg/µl); strip/tube 8, blank control. CL, control line; TL, test line.

Temperatures ranging from 60°C to 67°C with a 1°C interval were employed to conduct the MCDA reactions for the optimisation of the reaction temperature. As shown in **Figure 5**, 64°C was the optimal reaction temperature for the primer set, as the faster peak was obtained under this condition. Therefore, 64°C was subsequently used as the optimal temperature for the *M. pneumoniae* MCDA assay. In addition, the *M. pneumoniae* MCDA assay was conducted at 64°C for 10 min, 20 min, 30 min, and 40 min to reveal the optimal reaction time. As shown in **Figure 6**, it was concluded that the LoD level (determined by sensitivity analysis as 43 fg/µl) of the genomic DNA of *M. pneumoniae* could be detected only after amplification at 64°C for 40 min. Thus, a reaction temperature of 64°C and a reaction time of 40 min were employed in the following tests, and the whole diagnostic procedure, from template extraction to result reporting, was finished within 1 h (**Figure 2**).

### Sensitivity and specificity of the *M. pneumoniae* RT- MCDA assay

**Figure 7:**
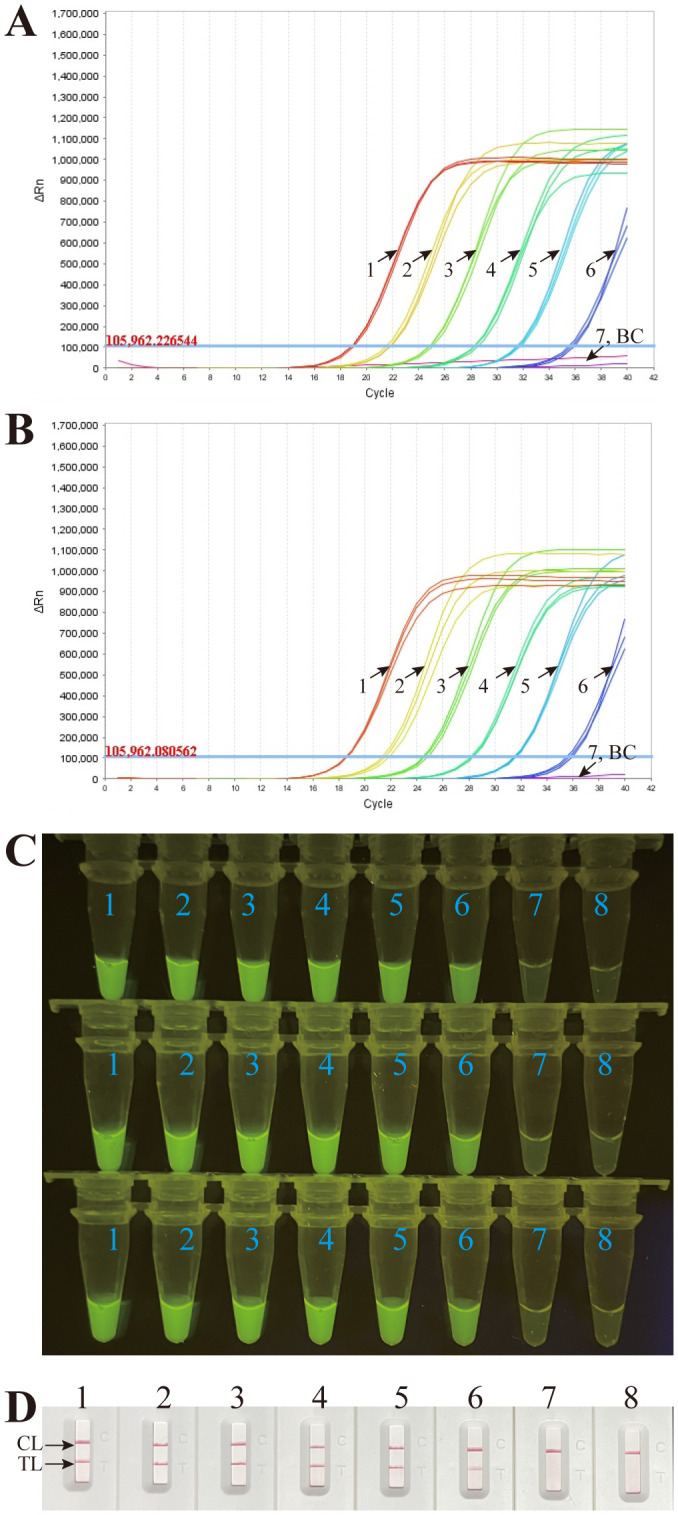
Sensitivity confirmation of the *M. pneumoniae* RT-MCDA assay. Sensitivity of the *M. pneumoniae* RT-MCDA assay was analysed using DNA templates extracted from serial dilutions (4.3 ng, 430 pg, 43 pg, 4.3 pg, 430 fg, 43 fg, and 4.3 fg/µl) of cultured *M. pneumoniae*. Three detection formats were used for amplification products analysis: **(A)** real-time fluorescence with 1 µl of template; **(B)** real-time fluorescence with 2 µl of template; **(C)** visual detection with the naked eye under blue light; and **(D)** an LFB test. The results of the three tests showed that the lowest detection limit of the *M. pneumoniae* RT-MCDA was 43 fg/µl. The sensitivity was also confirmed through visual detection with the naked eye under blue light **(C)** and an LFB test **(D)**. TL, test line; CL, control line.

To assess the sensitivity of the *M. pneumoniae* RT-MCDA assay, an RT-MCDA assay was performed with serial dilutions of genomic DNA templates from *M. pneumoniae* strains. The detection limits of the *M. pneumoniae* RT-MCDA assay with 1 µl or 2 µl of pure culture template were both 43 fg/µl (**Figures 7A, B**), which was in complete accordance with those observed with the naked eye under blue light (**Figure 7C**) and that when using an MCDA-LFB assay (**Figure 7D**). Considering the risk of carryover contamination, a reaction volume of 1 µl was recommended.

**Figure 8:**
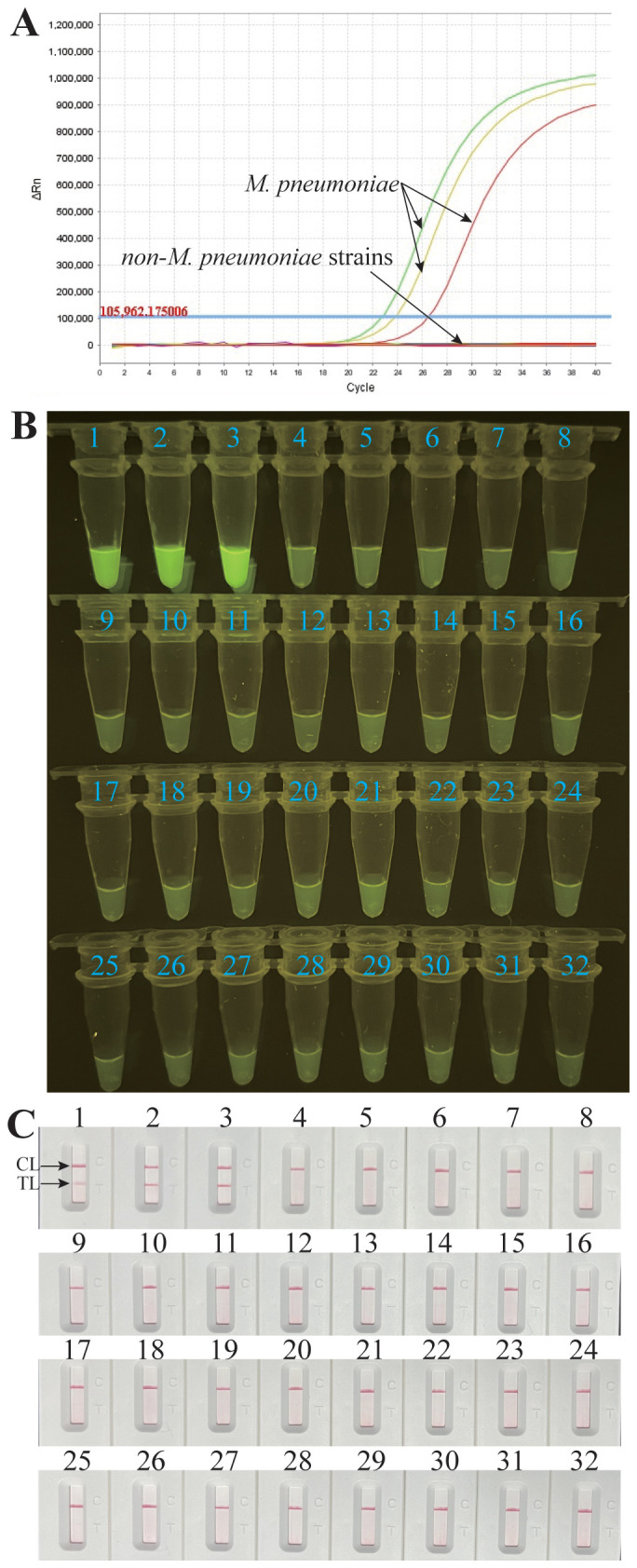
Analytical specificity of the *M. pneumoniae* RT-MCDA assay. DNA templates from three *M. pneumoniae* strains and 29 non-*M. pneumoniae* strains were tested using the *M. pneumoniae* RT-MCDA assay. No reactions were recorded with the non-*M. pneumoniae* strains. The generated fluorescence signals were recorded by the real-time PCR platform **(A)** and visually interpreted with the naked eye under blue light **(B)**. The results were also confirmed by an LFB test **(C)**. Signals/tubes/strips 1–3 represent the three *M. pneumoniae* strains, and the others represent the non-*M. pneumoniae* strains (**Table 2**). TL, test line; CL, control line.

To estimate the analytical specificity, the *M. pneumoniae* RT-MCDA assay was conducted using genomic DNA templates extracted from *M. pneumoniae* strains and non-*M. pneumoniae* strains (**Table 2**). When analysed using a real-time fluorometer, the positive results were only acquired from the reactions with DNA templates of *M. pneumoniae* strains; the non-*M. pneumoniae* strains were negative (**Figure 8A**). When observed under blue light, a light yellow colour was only observed with the products of three *M. pneumoniae* strains and not with those of the 29 non-*M. pneumoniae* pathogens (**Figure 8B**). When monitored by LFB, the three *M. pneumoniae* strains displayed two red lines at both TL and CL of LFB, whereas all the 29 non-*M. pneumoniae* pathogens displayed only one red line in CL of LFB (**Figure 8C**). All the above results proved that the specificity of the *M. pneumoniae* RT-MCDA assay was 100%.

### Application of the *M. pneumoniae* RT-MCDA assay in clinical specimens

**Figure 9:**
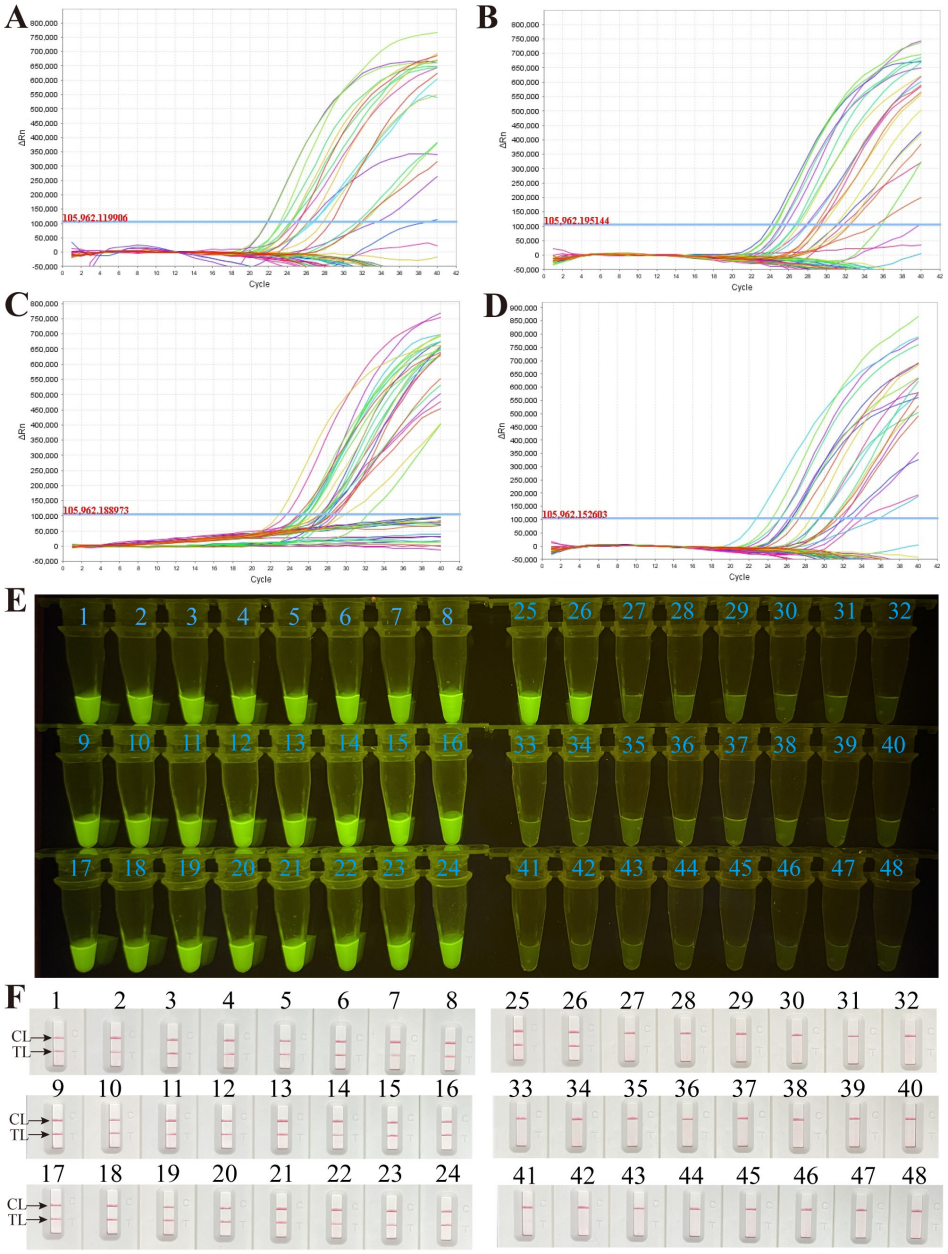
Application of the *M. pneumoniae* RT- MCDA assay in clinical specimens. DNA templates from 48 sputum samples were detected by the *M. pneumoniae* RT-MCDA assay. The produced fluorescent signals were documented by the real-time PCR system with 1 µl of template **(A)**, 3 µl of template **(B)**, 5 µl of template **(C)**, and 7 µl of template **(D)**, and visually interpreted with the naked eye under blue light **(E)**. The results were also confirmed by *M. pneumoniae*-MCDA-LFB **(F)**. Signals/tubes/strips 1–26 represent the results of the templates from the positive samples, and tubes/strips 27–48 represent the results of the *M. pneumoniae* negative samples. TL, test line; CL, control line.

To further validate the feasibility of the *M. pneumoniae* RT-MCDA assay in clinical application, the optimised process was used to detect the retrospectively collected DNA templates extracted from 48 sputum samples, which were previously detected using the real-time PCR method. Among the 48 samples, 26 tested positive for the *M. pneumoniae* RT-MCDA assay and 22 samples tested negative, regardless of whether the reaction volume was 1 µl, 3 µl, 5 µl, or 7 µl (**Figures 9A-D**). However, reactions with 1 µl and 3 µl of clinical sample template displayed more threshold results, which may be difficult to read with the naked eye, thus 5 µl of clinical sample template was selected for clinical evaluation analysis. Of note, the results obtained by the *M. pneumoniae* RT-MCDA assay (with 5 µl of template) (**Figures 9C, E**) were identical to that obtained by the *M. pneumoniae* MCDA-LFB assay (**Figure 9F**). In terms of the results obtained using the real-time PCR method, 26 positive specimens were previously determined positive and 22 specimens were negative. The complete accordance between the *M. pneumoniae* RT-MCDA assay and real-time PCR method implied the feasibility of the *M. pneumoniae* RT-MCDA assay in clinical practice.

## Discussion

In recent years, the global surge in macrolide resistance among *M. pneumoniae* strains has been particularly pronounced in China, where resistance levels have approached nearly 90%. This alarming trend has led to an increase in cases of severe pneumonia among patients who do not receive timely and effective treatment, posing a serious threat to public health (Kumar and Kumar, 2023; Chen et al., 2024). Furthermore, *M. pneumoniae* infections are characterised by non-specific clinical and imaging features, along with limited microbial diagnostic options, complicating the clinical diagnosis and increasing the likelihood of missing optimal treatment windows. This often exacerbates the patient’s disease burden (Kumar and Kumar, 2023). Consequently, there is a critical need for rapid and precise detection methods to identify and diagnose *M. pneumoniae* infections early and effectively.

Numerous isothermal amplification techniques have been developed for diagnosing pathogen infections, including nucleic acid sequence-based amplification, self-sustained sequence replication, strand displacement amplification, exponential amplification reaction, helicase-dependent amplification, recombinase polymerase amplification, single primer isothermal amplification, rolling circle amplification, loop-mediated isothermal amplification (LAMP), and cross priming amplification (Wang et al., 2016). However, these methods face technical challenges and do not entirely fulfill the point-of-care detection standards set by the World Health Organization (Srivastava and Prasad, 2023). MCDA is a relatively recent advancement in this area and addresses many limitations of earlier techniques (Wang et al., 2015). MCDA has gained broad application in fields such as basic research, medical diagnostics, and food safety (Qi et al., 2018; Boonbanjong et al., 2022; Li et al., 2022). Here, we introduce a novel real-time fluorescence MCDA method for *M. pneumoniae* that integrates real-time PCR technology with MCDA for efficient clinical detection. This approach uses a set of 10 sequence-specific primers that target 10 distinct regions (F1, F2, C1, C2, P1, P2, R1, R2, D1, and D2), ensuring high specificity (Wang et al., 2015, 2016). Critically, our technique employs the 5′ endonuclease recognition sites and fluorophore labelling to achieve precise cleavage of double-stranded DNA by the restriction enzyme *Nb.BsrDI*, releasing a fluorescence signal. Thus, the new real-time fluorescence MCDA method enables rapid multiplex detection and identification in a single isothermal step without the need for thermal cycling or subsequent analysis (Wang et al., 2015, 2016; Srivastava and Prasad, 2023). Additionally, the early high expression of the *CARDS* toxin gene during infection and the critical role of CARDS toxin in the pathogenesis of *M. pneumoniae* facilitate the *CARDS* toxin gene as a preferable biomarker for early and accurate *M. pneumoniae* detection (Chaudhry et al., 2016; Su et al., 2021). It was reported that detection assays targeting the *CARDS* toxin gene exhibited superior sensitivity compared with those targeting other genes such as the ATPase (Mp3 and Mp7) gene (Winchell et al., 2008). Thus, the real-time MCDA targeting the *CARDS* toxin gene in this study offers a more timely and efficient diagnosis, increasing early detection capabilities. In addition, a real-time PCR method targeting the P1 gene was used for clinical feasibility comparison.

In this study, we employed the restriction enzyme *Nb.BsrDI* and a modified primer, C1*, to detect MCDA reaction products through fluorescence. Additionally, another modified primer, D1^#^, and biotin-14-dCTP were used in a local feedback mechanism to monitor these products. We evaluated the sensitivity and specificity of these two methods through detailed analytical comparisons, finding both to be equally effective in detecting *M. pneumoniae*. Furthermore, the optimised real-time fluorescence MCDA detection process we developed can complete the identification of *M. pneumoniae* within 1 h, allowing for prompt treatment and control of the infection. Given its efficiency and reliability, the real-time MCDA detection method introduced in this study is highly recommended for the early diagnosis and management of *M. pneumoniae* infections.

The newly developed RT-MCDA assay demonstrated high sensitivity in detecting *M. pneumoniae*, with a LoD of 43 fg (approximately 47 copies) genomic DNA per reaction. This sensitivity is comparable with that achieved by real-time PCR (approximately 10 copies) (Morozumi et al., 2006) and slightly higher than that achieved by LAMP assays (50–600 fg) (Saito et al., 2005; Zhao et al., 2013; Wang et al., 2019; Xiao et al., 2022). A comparison of different methods for *M. pneumoniae* infection diagnosis is summarised in **Table 3** (Daxboeck et al., 2003; Templeton et al., 2003; Saito et al., 2005; Morozumi et al., 2006; Loens et al., 2010; Zhao et al., 2013; Wang et al., 2019; Xiao et al., 2022). **Table 3** conclusively shows that the RT-MCDA assay had superior sensitivity and timeliness but a relatively higher cost in *M. pneumoniae* detection; the LAMP-based assay displayed moderate sensitivity and timeliness as well as a relatively high cost; the real-time PCR method exhibited superior sensitivity and moderate timeliness and cost; the serological test displayed poor sensitivity but was superior in timeliness and cost; and the culture method showed poor sensitivity and timeliness but was the least costly. As a conclusion, the RT-MCDA assay was a superior option for *M. pneumoniae* detection with a further optimisation of sensitivity and cost. Moreover, the RT-MCDA assay exhibited no cross-reactivity with non-*M. pneumoniae* pathogens, achieving a specificity of 100%. To assess the clinical applicability of this method, we analysed 48 suspect samples, confirming *M. pneumoniae* in 26 cases (54.17%)—a rate consistent with that of real-time PCR. Although there were no significant statistical differences between the RT-MCDA method and conventional real-time PCR, further validation with a larger sample set is warranted. Additionally, the outcomes of the *M. pneumoniae* RT-MCDA assay are not only trackable through a real-time fluorescence monitoring system but can also be visually confirmed under blue light, significantly reducing the contamination risks typically associated with opening the reaction container. Given its robust performance and operational flexibility, this novel method holds promise for its widespread use in clinical diagnostics, field testing, and resource-constrained environments.

**Table 3:** Sensitivity, time, and cost comparison of different methods for *M. pneumoniae* detection.

Methods	LoD	Time (min)	Cost (RMB)
RT-MCDA assay	43 fg (~47 copies)/reaction	~40 min	~60
LAMP-based assay	50~600 fg (55~660 copies)/reaction	40~60 min	~60
Real-time PCR	~10 copies/reaction	~90 min	~50
Serological test	17 U/ml	~15 min	~30
Culture	10^5^ cfu/ml	2~6 week	~20

In summary, this study successfully developed a real-time MCDA detection method for *M. pneumoniae* that offers timely, simple, and reliable testing. This novel assay represents a promising tool for the rapid, sensitive, and specific identification of *M. pneumoniae* infections and stands as a viable option for increasing diagnostic capabilities.

## Data availability statement

The original contributions presented in the study are included in the article/supplementary material. Further inquiries can be directed to the corresponding authors.

## Ethics statement

The studies involving human participants were reviewed and approved by the ethic committee of Capital Institute of Pediatrics (Ethical approval number: SHERLLM2021010). The patients/participants [legal guardian/next of kin] provided written informed consent to participate in the study.

## Author contributions

FX: Writing – original draft, Conceptualization, Data curation, Formal Analysis, Investigation, Methodology, Validation, Writing – review & editing. YZ: Data curation, Formal Analysis, Writing – original draft. WX: Conceptualization, Writing – original draft. JF: Project administration, Writing – review & editing. XH: Project administration, Writing – review & editing. NJ: Formal Analysis, Writing – review & editing. CS: Software, Writing – review & editing. ZX: Data curation, Writing – original draft. BZ: Conceptualization, Resources, Writing – review & editing. JZ: Formal Analysis, Supervision, Writing – review & editing. YW: Data curation, Formal Analysis, Funding acquisition, Methodology, Project administration, Resources, Software, Writing – review & editing. LM: Project administration, Resources, Supervision, Validation, Writing – review & editing.
